# Not One Pandemic: A Multilevel Mixture Model Investigation of the Relationship Between Poverty and the Course of the COVID-19 Pandemic Death Rate in the United States

**DOI:** 10.3389/fsoc.2021.629042

**Published:** 2021-10-22

**Authors:** Holmes Finch, Maria E. Hernández Finch, Katherine Mytych

**Affiliations:** Educational Psychology, Ball State University, Muncie, IN, United States

**Keywords:** multilevel mixture model, COVID-19, United States, poverty, demographic characteristic

## Abstract

The COVID-19 pandemic, which began in China in late 2019, and subsequently spread across the world during the first several months of 2020, has had a dramatic impact on all facets of life. At the same time, it has not manifested in the same way in every nation. Some countries experienced a large initial spike in cases and deaths, followed by a rapid decline, whereas others had relatively low rates of both outcomes throughout the first half of 2020. The United States experienced a unique pattern of the virus, with a large initial spike, followed by a moderate decline in cases, followed by second and then third spikes. In addition, research has shown that in the United States the severity of the pandemic has been associated with poverty and access to health care services. This study was designed to examine whether the course of the pandemic has been uniform across America, and if not how it differed, particularly with respect to poverty. Results of a random intercept multilevel mixture model revealed that the pandemic followed four distinct paths in the country. The least ethnically diverse (85.1% white population) and most rural (82.8% rural residents) counties had the lowest death rates (0.06/1000) and the weakest link between deaths due to COVID-19 and poverty (*b* = 0.03). In contrast, counties with the highest proportion of urban residents (100%), greatest ethnic diversity (48.2% nonwhite), and highest population density (751.4 people per square mile) had the highest COVID-19 death rates (0.33/1000), and strongest relationship between the COVID-19 death rate and poverty (*b* = 46.21). Given these findings, American policy makers need to consider developing responses to future pandemics that account for local characteristics. These responses must take special account of pandemic responses among people of color, who suffered the highest death rates in the nation.

## Introduction

Beginning in late 2019, a novel Coronavirus, later named COVID-19, emerged in Wuhan, China. This virus spread quickly in parts of China, and soon moved to other nations in the region, eventually spreading across the world by the early spring of 2020. The course of this pandemic has varied greatly across nations, with some experiencing steep spikes in the infection and death rates early in 2020 followed by a sharp decline in the summer, and then a rebound in the fall of 2021 (e.g., Italy), with others having consistently lower rates of both outcomes throughout this time period (e.g., Singapore, Korea, and Hong Kong). The United States experienced a somewhat unique course of the pandemic, with an increase in cases through the early spring, followed by a decline in late spring, and then a large spike in case numbers through the summer and into the fall (New York Times, 2020), with much spatial variation in where the spikes occurred at different points in time. This uneven course of the pandemic in the United States hints at its variable course in different parts of the country.

Research, as well as popular media outlets, have reported a link between poverty, ethnicity, and the severity of the pandemic’s effects on Americans (Adhikari, et al., 2020; Finch and Hernández Finch, 2020; Goldstein, 2020; Koma, et al., 2020; USA Today, 2020). It is also known that COVID-19 is particularly dangerous for individuals with comorbidities such as diabetes, heart disease, and pulmonary illnesses, all of which tend to be more prevalent in under-resourced communities (Oates, et al., 2017; Williams, et al., 2010; Elo, 2009; Adler and Rehkopf, 2008; Braverman, et al., 2005; Lutfey and Freese, 2005; Link and Phelan, 1995). The impact of these relationships among various COVID-19 and other diseases is exacerbated by the fact that individuals living in under-resourced communities may also lack access to high quality health care (James, et al., 2008; Shi and Stevens, 2005; Lorant, et al., 2002), thereby making the consequence of catching COVID-19 even more severe. The current work follows in the line of research investigating the course of the COVID-19 pandemic over time and for identifying the underlying pattern of spread across the world (Maleki, et al., 2020a; Mahmoudi, et al., 2021b; Maleki, et al., 2020b; Mahmoudi, et al., 2020; Mahmoudi et al., 2021a).

## Study Goals

The primary goal of this study was to ascertain whether there existed multiple subsets of counties within the United States with respect to the relationship between poverty and the number of COVID-19 cases, and in terms of the trajectory of the case rate over time. A second goal, assuming that multiple such latent classes were found, was to compare them on a variety of demographic, income, and health outcome variables. Taken together, these two strains of investigation were designed to characterize the nature of the pandemic and its course in the United States throughout January, 2021, in an attempt to better understand how it may, or may not, have impacted different subgroups within the population differently. This study contributes a unique perspective to the COVID-19 literature by identifying differing change trajectories in the course of the pandemic based upon geography within the United States, and by characterizing these trajectories with respect to a wide array of demographic, economic, educational, and health variables. This comprehensive examination into the course of the COVID-19 pandemic will provide researchers and policy makers with insights that should help drive future research as well as efforts to mitigate the negative impacts of the pandemic in the most severely impacted communities.

## Methods

### Data Sources

Several sources were used to obtain the data used in this study. The numbers of confirmed COVID-19 cases and deaths were downloaded from the New York Times at https://github.com/nytimes/covid-19-data on June 8, 2021. A full description of the dataset appears on the data website, with a brief description. County level data were collected from state and local health departments, with the first case in the set being January 21, 2020, and the last case being February 1, 2021, which corresponds to a period immediately prior to the wide scale uptake of COVID-19 vaccinations in the United States. The FIPS code for each county was included in the dataset, which allowed for it to be merged with other datasets that also include this county identifier.

Data on poverty came from the poverty solutions initiative (PSI) at the University of Michigan (https://poverty.umich.edu/about/). Specifically, the Index of Deep Disadvantage, hereafter referred as the poverty index, or index, was used as one of the two primary independent variables in the statistical modeling, which is described below. The poverty index data for each county in the United States, along with the county FIPS code were included in the dataset used in this study. Merging of the data by FIPS code allowed for the matching of poverty index values to the case rates in the NY Times COVID-19 data. The Index of Deep Disadvantage is described in full detail at (https://poverty.umich.edu/files/2020/01/IDD-Technical-documentation-1.pdf).

In addition to the COVID-19 case rates and the poverty index, additional variables describing a variety of income, health, unemployment, and mobility factors for counties in the United States were also used in this study. A full list of these variables, along with their sources appears in Table 1. The county demographic data were obtained from the U.S. Census Bureau, education outcomes from the U.S. Department of Education, health indicators from the Centers for Disease Control and Prevention (2020), and income and unemployment data from the U.S. Department of Labor. The indicators of the relative urban and rural natures of the counties were gathered by the Department of Education, and the mobility data were obtained from the website for the Community Mobility Reports project at https://www.google.com/covid19/mobility/.

**TABLE 1:** Variables used in the analyses.

Variable	Source
COVID-19 cases	New York Times COVID-19 project
COVID-19 deaths	New York Times COVID-19 project
Index of Deep Disadvantage	University of Michigan Poverty Solutions Initiative
% less than 18	United States Census Bureau
% 65 and over	United States Census Bureau
% White	United States Census Bureau
% African American	United States Census Bureau
% Latina	United States Census Bureau
% American Indian/Alaska Native	United States Census Bureau
% Asian	United States Census Bureau
% Native Hawaiian/Pacific Islander	United States Census Bureau
% Not proficient in English	United States Census Bureau
High school graduation rate	United States Department of Education
% with access to exercise	United States Centers for Disease Control
% smokers	United States Centers for Disease Control
Age-adjusted death rate	United States Centers for Disease Control
Years of potential life lost	United States Centers for Disease Control
% physically inactive	United States Centers for Disease Control
Infant mortality	United States Centers for Disease Control
Child mortality	United States Centers for Disease Control
Mean number of unhealthy days	United States Centers for Disease Control
% Adults with obesity	United States Centers for Disease Control
% Adults with diabetes	United States Centers for Disease Control
% Fair or poor health	United States Centers for Disease Control
% Vaccinated	United States Centers for Disease Control
% Uninsured	United States Centers for Disease Control
Average daily particulate matter 2.5	United States Centers for Disease Control
80th percentile income	United States Department of Labor
20th percentile income	United States Department of Labor
Income ratio	United States Department of Labor
Median household income	United States Department of Labor
Median household income as percent of state total	United States Department of Labor
% Enrolled free lunch	United States Department of Education
% Unemployment	United States Centers for Disease Control
% Severe housing cost burden	United States Centers for Disease Control
% Homeowners	United States Centers for Disease Control
% Severe housing problems	United States Centers for Disease Control
Overcrowding	United States Centers for Disease Control
%Food insecure	United States Centers for Disease Control
Inadequate facilities	United States Centers for Disease Control
% Limited access to healthy foods	United States Centers for Disease Control
Food environment index	United States Centers for Disease Control
2013 Rural-Urban code	United States Department of Education
Urban influence code	United States Department of Education
Metro	United States Department of Education
Transit stations	Community Mobility Report
Retail/recreation	Community Mobility Report
Workplaces	Community Mobility Report
Residential	Community Mobility Report
Grocery/pharmacy	Community Mobility Report
Parks	Community Mobility Report

### Variables

The outcome variable of interest in this study was the cumulative numbers of deaths due to COVID-19 cases for each county in the United States. When considering the results of this study it is important to keep in mind that only deaths that have been confirmed by state and local health authorities are included here. The two independent variables included in the mixture multilevel regression model (described below) were time, operationalized as the week of the pandemic, and the poverty index. COVID-19 case data were combined by week, and week was numbered from 1 to 38.

The poverty index was developed by researchers in the PSI using principal components analysis (PCA). More specifically, the index was the first principal component obtained using PCA involving five features that have been demonstrated to be associated with poverty and disadvantage (Robles et al., 2019). The researchers reported that this first component accounted for more than 60% of the variance in the set of variables. The weights obtained from the PCA were then applied to the set of constituent variables in order to obtain an index score for each community. After obtaining the index scores, the researchers undertook sensitivity analyses in order to ensure that the index was, in fact, reflecting relative disadvantage as its intent. The results of these sensitivity analyses did indeed support the validity of the index, as reported in Robles, Simington, and Shaefer (https://poverty.umich.edu/files/2020/01/IDD-Technical-documentation-1.pdf). The index is scaled such that higher values indicate a higher degree of advantage; i.e., relatively more prosperous communities. Thus, lower scores were associated with communities experiencing greater economic disadvantage.

Several variables were used in constructing the poverty index. These include, Chetty and Hendren (2018) estimate of social mobility, life expectancy, percent of residents living below the poverty line, percent of residents living in deep poverty, and the percent of low birth weights. In addition, the PSI also collected other variables that might be associated with poverty, including whether the community was urban or not, and percent of residents with less than a high school diploma. Communities were defined as urban-based on a definition used by the National Center for Health Statistics, and appearing at this website: https://www.cdc.gov/nchs/data_access/urban_rural.htm. Specifically, urban counties contained a metropolitan statistical area (MSA) of 1 million or more individuals, or that have the entire population contained within the largest principal city of the MSA, or contain at least 250,000 in habitants of any principal city of the MSA. In addition, urban counties were also defined as those with a population of 1,000,000 or higher but which did not meet the aforementioned standards, or those with MSAs of 250,000–999,999.

## Data Analysis

### Multilevel Mixture Model

The primary data analytic strategy used in this study was a multilevel mixture model with number of deaths due to COVID-19 per 1,000 residents serving as the dependent variable, and week and the poverty index as the independent variables. The level-2 cluster indicator variable was county. Solutions from 2 to 5 latent classes were fit to the data. In order to determine the appropriate number of classes to retain, several indices were used, including AIC, BIC, sample size adjusted BIC (aBIC), and entropy. These statistics have been shown to be effective tools for determining the number of latent classes to retain (Wang et al., 2017; Nylund et al., 2007). With respect to AIC, BIC, and aBIC, lower values indicate better fit after adjusting for model complexity, meaning that the model with the lowest value is considered to have the best fit. In contrast, the model with the largest entropy (which ranges from 0 to 1) was considered to yield the best fit, with values in excess of 0.8 indicating a well separated set of latent classes (Celeux and Soromenho, 1996).

A random intercept multilevel model with week and the poverty index as independent variables was fit into the data within the mixture framework. Therefore, the latent classes were differentiated with respect to the magnitudes of the coefficients for these two variables, as well as the estimates of the error and intercept variances. In addition to the model including both week and the poverty index, a null model with no independent variables was fit into the data within each latent class in order to obtain the variance estimates necessary to calculate the intraclass correlation (ICC). The ICC reflects the proportion of variation in the outcome variable (number of cases) that was associated with the level-2 variable (county). Higher levels of the ICC indicate that a greater proportion of the dependent variable variance was associated with the counties.

### Discriminant Analysis

In order to further explore the nature of the latent classes defined above, statistics for a number of additional variables were compared across the latent classes. Given the large number of such variables, discriminant analysis (DA) was used, and variables with structure coefficients greater than 0.4 were identified as being associated with the discriminant functions. Discriminant functions that were statistically significant based on the Wilks' Lambda statistic, and that also accounted for more than 10% of the variance in the data were deemed to be worth further discussion. This latter criterion was selected due to the large sample size, all of the hypothesis tests for the discriminant functions were statistically significant. Thus, it was decided that the function also needed to account for at least 10% of the variance in the differences across the latent classes in order to be worth further discussion. The variables within each set were standardized prior to the fitting of the DA models.

## Results

### Determining the Number of Latent Classes

The information indices all indicated that the 4 class model was optimal, as seen in Supplementary Table S1. The AIC, BIC, and aBIC were all smallest for the 4 class solution, and its entropy was the second largest with classes. In addition, the average latent class probabilities of the most likely latent class were 0.91 or higher for each of the classes (Supplementary Table S2). This result suggests that the 4-class solution was very stable, providing additional confidence in its viability. The proportion of counties in each latent class appear in Supplementary Table S3. Classes 1 and 3 were the largest, accounting for 33 and 39% of the counties, respectively. Class 4 was the smallest, account for 3% of the counties, and class 3 consisted of 25% of American counties.

### Model Parameter Estimates by Latent Classes

Supplementary Table S3 also displays the GCM model parameter estimates for each latent class. In addition, the table includes the mean of the poverty index, and the ICC associated with county for each class. For all of the classes, the relationship between week and the number of deaths per 1,000 population was positive, indicating that the number of deaths increased in value over time. In addition, for each of the classes, the relationship between the poverty index and number of cases was negative, indicating that poorer counties had a higher number of cases per 100,000 residents. Overall, the coefficient linking time to the number of deaths per 1,000 was significantly different for all pairwise comparisons of the classes (Table 2). This result indicates that the latent classes did indeed differ in terms of the course of the pandemic over time. The coefficient for the poverty index also differed significantly between all pairs of classes, except for 1 and 3.

**TABLE 2:** *t*-statistics for comparisons of mixed effects parameter estimates between pairs of latent classes.

Variable	1 v 2	1 v 3	1 v 4	2 v 3	2 v 4	3 v 4
Week	−27.57*	−19.74*	−30.84*	21.08*	−28.50*	−30.37*
Index	−17.00*	−2.69	−4.61*	15.79*	−3.55*	−4.56*

Bonferroni critical value = 2.88.

* Structure coefficient greater than or equal to 0.4.

**FIGURE 1:**
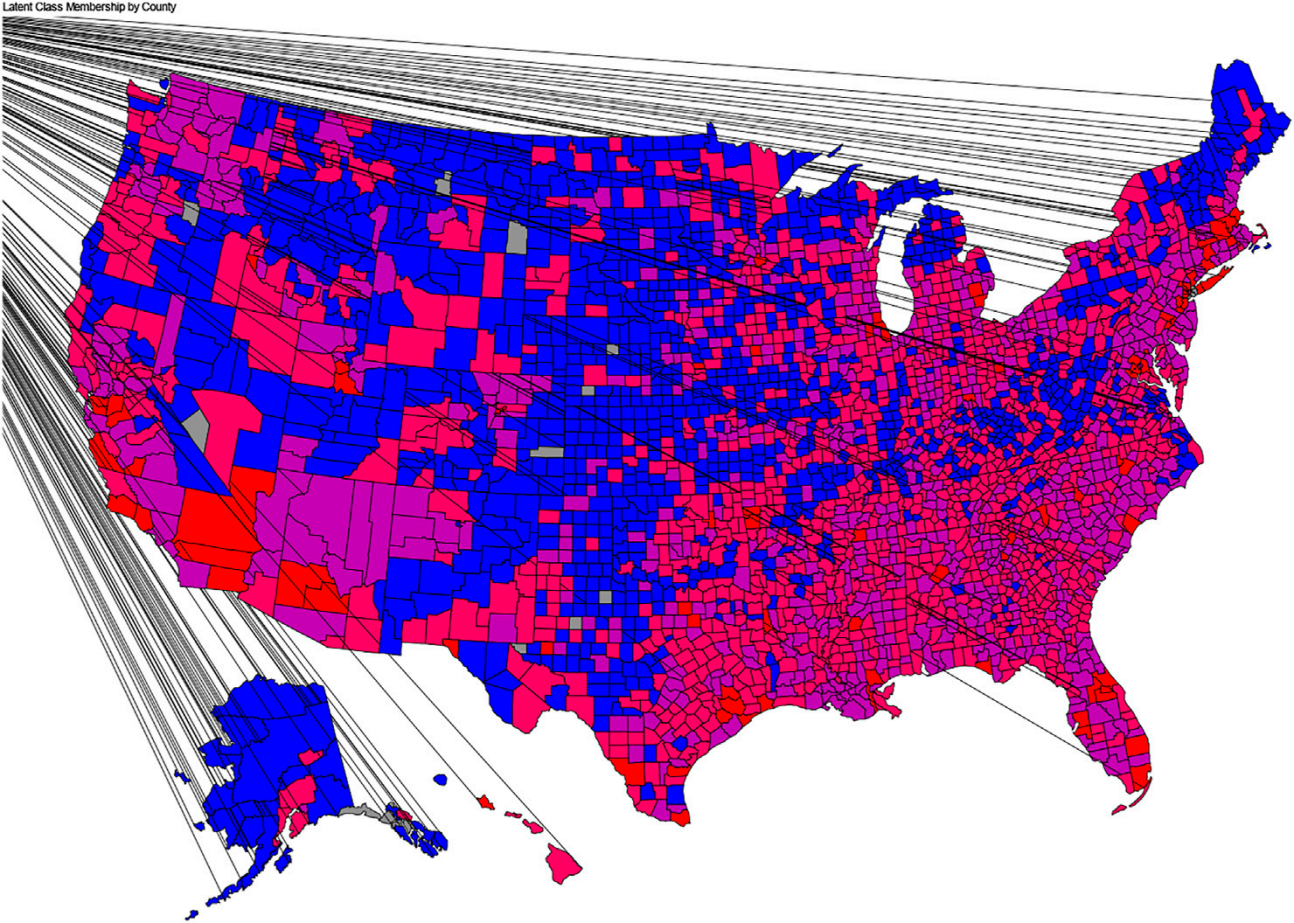
Map of Latent Class membership by county.

The distribution of latent classes by counties appears in Figure 1. Class 1 was centered primarily in the middle of the United States, with some pockets in the northern portions of each coast. Class 4 was centered primarily in urban areas along the east and west coasts, as well as in cities such as Chicago, Atlanta, Detroit, Houston, and Dallas. Classes 2 and 3 were found throughout the United States, with particularly concentrations in the southeast and midwest.

In addition to the overall results described above, there were a number of class specific estimates of interest. The results in Table 2, Supplementary Table S3 reveal for the counties in Class 1, the relationship between poverty and the death rate was the weakest, and the rate of increase in the number of deaths over time was the slowest. In addition, Class 1 had the highest index value, indicating that its counties were the wealthiest on average. Latent Class 2, which was the second poorest of the four, exhibited the second fastest rate of growth in the number of deaths per week, and had the second strongest relationship between the index and number of COVID-19 cases. The counties in latent Class 3 manifested the second weakest relationship between the poverty index and the number of COVID-19 deaths per 1,000 across the 4 classes. Class 3 also had the second fastest growth rate in the number of deaths over time, and was the poorest. Finally, Class 4 had the strongest relationship between poverty and deaths, and the fastest growth rate in the number of deaths per week. It also consisted of the second wealthiest group of counties. With respect to the ICC, Class 3 exhibited the largest value for this sample (0.49), followed by Class 4 (0.35), with Classes 1 (0.17), 3 (0.16), and 4 (0.15) had comparable ICCs with that for Class 2 (0.11) being slightly lower. These results indicate that county accounted for between 11 and 17% of the variance in the number of deaths per 1,000.

### Death Rate

The deaths per 1,000 rate was compared across the latent classes using a nested ANOVA, with county nested in latent class. There was a statistically significant difference among the latent class means, with latent class accounting for 15.3% of the variance in the death rate per 1,000 (F3,78250 = 4704.19, p < 0.001, η2 = 0.153 ). The deaths per 1,000 means by latent class appear in Table 3. The Tukey-b post hoc test results revealed that the means were all statistically significant from one another, with Class 4 having the highest value, Class 2 the second highest, and Class 1 the lowest death rate per 1,000 residents.

**TABLE 3:** Mean (standard error) of deaths/1,000 residents and deaths per number of cases by latent class.

Latent class	Mean (standard error)	95% Confidence interval
**Deaths per 1,000 residents**		
1	0.060 (0.001)	0.058, 0.062
2	0.303 (0.003)	0.297, 0.308
3	0.178 (0.002)	0.174, 0.182
4	0.327 (0.0.01)	0.307, 0.348
**Deaths per COVID-19 cases**		
1	0.020 (0.0004)	0.019, 0.021
2	0.032 (0.0002)	0.031, 0.033
3	0.027 (0.0003)	0.026, 0.028
4	0.035 (0.0001)	0.034, 0.036

ANOVA results showed that there was also a statistically significant difference in number of deaths per number of cases across the latent classes, with an effect size of 0.007 (F3,78250 = 181.755, p < 0.001, η2 = 0.007). The means, standard errors, and 95% confidence intervals for the number of deaths per number of cases appear in Table 3. Although statistically significantly different, the mean death rates per number of cases were within 0.015 of one another, with Class 4 having the highest value, and Class 1 the lowest. The relative close proximity of these values, coupled with the low effect size (latent classes accounted for less than 1% of the variance in the deaths per number of cases) suggest that there was little practical difference across the classes for this variable.

### Demographic Variables

The first DA function for the comparison of the latent class means for the demographic variables appearing in Table 4 were statistically significant, and accounted for at least 10% of the variance in the demographic variables. The structure coefficients for the two functions appear in Table 4, with coefficients of 0.4 or more denoted with an asterisk. With respect to function 1, the variables 65% and over, white%, African American%, %Asian%, and percentage not proficient in English all had values of 0.4 or more.

**TABLE 4:** Discriminant analysis structure coefficients for demographic variables by latent class.

	Function 1 (84.1%)
% less than 18	−0.19
% 65 and over	0.66^a^
% White	0.59^a^
% African American	0.46^a^
% Latina	−0.26
% American Indian/Alaska Native	0.10
% Asian	−0.52^a^
% Native Hawaiian/Pacific Islander	−0.10
% Not proficient in English	−0.42^a^
High school graduation rate	0.26

a Structure coefficient greater than or equal to 0.4.

The means for each variable within the demographic set by latent class appear in Table 5, with variables having structure coefficients denoted with an asterisk. These results reveal that the counties in Class 1 had the highest percent of residents who were 65 years or older, and who were white, and the lowest percent who were African American, Latina, Asian, and not proficient in English. In contrast, Class 4 had the lowest percent of residents who were white, and the highest percent who were Latina, Asian, and not proficient in English, and the lowest percent who were 65 years or older. Latent Classes 2 and 3 had higher percentages of residents who were 65 and over, and white, than did Class 4, but lower than that of Class 1. In addition, Classes 2 and 3 had lower percentages of residents who were Latina, Asian, and not proficient in English than did Class 4.

**TABLE 5:** Means for demographic variables by latent class.

Variable	Class 1	Class 2	Class 3	Class 4
% less than 18	21.5	22.7	22.2	22.9
% 65 and over^a^	21.7	16.7	18.5	15.1
% White^a^	84.9	67.9	74.8	51.8
% African American^a^	2.5	14.3	11.6	16.4
% Latina	7.5	11.9	8.9	22.6
% American Indian/Alaska Native	2.9	1.7	2.0	1.0
% Asian^a^	0.8	2.7	1.2	6.7
% Native Hawaiian/Pacific Islander	0.1	0.2	0.1	0.2
% Not proficient in English^a^	1.0	2.5	1.5	5.5
High school graduation rate	90.2	87.1	88.6	85.2

a Discriminant analysis structure coefficient greater than or equal to 0.4.

### Health Indicators

One discriminant function was found to be both statistically significant and accounted for 83.3% of the variance in the group differences across the latent classes for the set of health indicators appearing in Table 6. The variables with structure coefficients greater than 0.4 are denoted with an asterisk. The means for all of the health indicators by latent class appear in Table 7. Class 4 had the highest percent of individuals with access to regular exercise, the lowest percent of smokers, the lowest age-adjusted death rate, the lowest years of potential life lost, the lowest percent of individuals who were physically inactive, and the lowest percent of residents who were obese, or who had diabetes. Class 1 had the least access to exercise, with Class 3 having the next lowest such percent, and Class 2 the second highest. Class 3 had the highest years of potential life lost, followed by Classes 1 and 4. With regard to percent of smokers, percent physically inactive, percent obese, and with diabetes, Classes 1,2, and 3 had similar results. Finally, Class 1 had the lowest average daily particulate matter values.

**TABLE 6:** Discriminant analysis structure coefficients for health outcomes by latent class.

	Function 1 (83.3%)
% with access to exercise	0.67^a^
% smokers	−0.49^a^
Age-adjusted death rate	−0.42^a^
Years of potential life lost	−0.40^a^
% physically inactive	−0.40^a^
Infant mortality	−0.38
Child mortality	−0.30
Mean number of unhealthy days	−0.36
% Adults with obesity	−0.47^a^
% Adults with diabetes	−0.42^a^
% Fair or poor health	−0.15
% Vaccinated	0.19
% Uninsured	0.04
Average daily particulate matter 2.5	−0.41^a^

a Structure coefficient greater than or equal to 0.4.

**TABLE 7:** Means of health outcome variables by latent class.

	Class 1	Class 2	Class 3	Class 4
% with access to exercise*	57.34	72.77	61.31	90.66
% smokers*	16.98	17.14	18.38	14.79
Age-adjusted death rate*	396.42	391.12	433.40	329.22
Years of potential life lost*	8582.00	8070.18	8940.85	6774.57
% physically inactive*	27.07	26.29	28.50	22.75
Infant mortality	9.01	6.53	7.14	5.78
Child mortality	72.94	56.74	63.35	49.84
Mean number of unhealthy days	3.86	3.96	4.15	3.70
% Adults with obesity*	32.28	32.59	34.04	28.08
% Adults with diabetes*	11.58	11.74	13.07	9.73
% Fair or poor health	16.67	17.98	19.11	16.99
% Vaccinated	37.99	46.29	43.18	46.51
% Uninsured	12.83	13.33	14.06	13.10
Average daily particulate matter 2.5*	7.99	8.82	9.57	10.15

* Structure coefficient greater than or equal to 0.4.

### Income

For the income and poverty variables, one discriminant function was found to be statistically significant, and accounted for 84.5% of the variation in the class differences across the variables. The variables with structure coefficients greater than 0.4 were 80th percentile income, 20th percentile income, median household income, and median household income as percent of state total (Table 8). The means for all of the income variables appear in Table 9. Class 4 had the highest 80th percentile income, 20th percentile income, median household income, and median household income as percent of the state total. The lowest values for these variables belonged to Classes 1 and 3, with Class 2 having the second highest values.

**TABLE 8:** Discriminant analysis structure coefficients for income/employment variables by latent class.

	Function 1 (84.5%)
80th percentile income	0.77^a^
20th percentile income	0.40^a^
Income ratio	0.29
Median household income	0.62^a^
Median household income as percent of state total	0.59^a^
% Unemployment	−0.03

a Structure coefficient greater than or equal to 0.4.

**TABLE 9:** Means for income/employment variables by latent class.

	Class 1	Class 2	Class 3	Class 4
80th percentile income^a^	93797.26	108903.84	94792.03	131401.21
20th percentile income^a^	22275.40	24552.83	21275.50	27706.65
Income ratio	4.33	4.60	4.62	4.85
Median household income^a^	50811.26	58411.63	50667.08	68509.26
Median household income as percent of state total^a^	85.30	98.15	87.72	106.47
% Unemployment	4.08	4.05	4.25	4.06

a Structure coefficient greater than or equal to 0.4.

### Housing and Food Security

The DA results for the housing and food security variables revealed that function 1 was statistically significant, and accounted for 88.1% of the variance in the housing/food variables. Results in Table 10 show that the severe housing cost burden percentage, homeowners percentage, severe housing problems percentage, and population density were associated with function 1.

**TABLE 10:** Discriminant analysis structure coefficients for housing/food variables by latent class.

	Function 1 (88.1%)
% Severe housing cost burden	−0.69^a^
% Homeowners	0.65^a^
% Severe housing problems	−0.58^a^
Overcrowding	−0.22
%Food insecure	−0.10
Inadequate facilities	0.18
% Limited access to healthy foods	0.20
Food environment index	−0.05
Population density	0.64^a^

a Structure coefficient greater than or equal to 0.4.

The means of the housing and food security variables appear in Table 11. Residents of counties in Class 1 had the lowest percent of residents with a severe housing cost burden, or severe housing problems, population density, and with the highest rate of homeownership. In contrast, the highest severe housing burden, severe housing problem, population density, and lowest homeownership rates were found in Class 4. Classes 2 and 3 had similar values for homeowners percentage. Class 3 had the second lowest percentage of residents with severe housing cost burden, severe housing problems, and population density.

**TABLE 11:** Means for housing/food variables by latent class.

	Class 1	Class 2	Class 3	Class 4
% Severe housing cost burden*	11.64	15.37	12.21	16.05
% Homeowners*	75.06	68.30	70.78	61.66
% Severe housing problems*	12.21	15.37	14.19	19.43
Overcrowding	2.07	2.64	2.46	3.66
%Food insecure	12.34	13.33	14.10	12.54
Inadequate facilities	1.47	1.00	1.10	0.91
% Limited access to healthy foods	9.73	7.60	7.32	5.85
Food environment index	7.52	7.53	7.41	7.84
Population density*	13.85	169.97	50.75	751.40

* Structure coefficient greater than or equal to 0.4.

### Urban/Rural

Another set of variables to be examined with regard to the latent classes were the urban/rural indicators. One of the discriminant functions was both statistically significant, and accounted for 92.3% of the variance in the separation among the classes. Based on the results in Table 12, all three urban/rural variables had structure coefficients of 0.4 or more. The means for these variables by latent class appear in Table 13. These results reveal that the counties in Class 1 were the most rural (highest values on rural-urban and urban influence codes, lowest metro value). In contrast, Class 4 included the most urban counties, followed by Class 2. Latent Class 3 was the second least urban based on these variables.

**TABLE 12:** Discriminant analysis structure coefficients for urban/rural variables by latent class.

	Function 1 (92.3%)
Rural-Urban code	0.96^a^
Urban influence code	0.86^a^
Metro	−0.67^a^

a Structure coefficient greater than or equal to 0.4.

**TABLE 13:** Means for urban/rural variables by latent class.

	Class 1	Class 2	Class 3	Class 4
Rural-Urban code*	6.69	2.97	4.48	1.37
Urban influence code*	7.30	2.89	4.51	1.33
Metro*	0.16	0.69	0.39	1.00

* Structure coefficient greater than or equal to 0.4.

### Mobility Changes

One discriminant function was statistically significant and accounted for 97.2% of the variance in separation among the latent classes. Based on Table 14, changes for all of the mobility were associated with between the class differences. The means for the mobility changes appear in Table 15. Class 4 had the greatest decrease in train station, retail/recreation, workplace, and grocery/pharmacy use. Class 4 also had the largest increase in residential use. Counties in Class 3 experienced the smallest changes in use of transit stations, retail/recreation, and grocery/pharmacy, and along with Class 1 the smallest change in workplace mobility.

**TABLE 14:** Discriminant analysis structure coefficients for mobility changes variables by latent class.

	Function 1 (97.2%)
Transit stations	0.86^a^
Retail/Recreation	0.70^a^
Workplaces	0.64^a^
Residential	−0.61^a^
Grocery/pharmacy	0.51^a^
Parks	0.56^a^

a Structure coefficient greater than or equal to 0.4.

**TABLE 15:** Means for mobility change variables by latent class.

	Class 1	Class 2	Class 3	Class 4
Transit stations	−28.78	−19.64	−14.54	−36.98
Retail/recreation	−14.68	−13.14	−9.59	−19.66
Workplaces	−22.53	−26.08	−23.32	−31.64
Residential	6.37	8.02	6.64	9.84
Grocery/pharmacy	−3.27	−4.44	−2.88	−9.06
Parks	9.06	19.70	10.42	6.24

### Relationship Between Income, Poverty, and Case Rates Within Latent Classes

Finally, the within class correlation coefficients for the relationships between the number of COVID-19 deaths per 1,000 residents with median household income, as well as percent of residents living in poverty, appear in Table 16. Given the large sample sizes, all of these correlations were statistically significant at *α* = 0.05. Therefore, interpretation will focus on the magnitudes of the coefficients, with Cohen’s (1988) guidelines used to characterize them as small (0.1–0.3), moderate (0.31–0.5), and large (0.51–1.00). For latent Class 1, the relationships between the case rates and each of the variables in Table 16 were negligible in size. Similarly, for Class 2 the correlations for 80th percentile income and median income were also in the negligible range. On the other hand, higher values of % enrolled free lunch, % unemployed, and % poverty were associated with higher death rates for counties in this class. For the other two classes, the higher the median household income, 80th percentile income, and 20th percentile income for a county, the lower the number of deaths, with the strongest such relationships occurring for Class 4. In addition, counties with a larger percent of residents who were unemployed, living in poverty, or whose children were on free/reduced lunch at school had more deaths per 1,000 residents.

**TABLE 16:** Correlation coefficients between the number of deaths per 1,000 residents and income and poverty variables within each latent class.

Variable	Class 1	Class 2	Class 3	Class 4
Median income	−0.02	−0.09	−0.19	−0.27
% Enrolled free lunch	0.06	0.18	0.25	0.18
% Unemployed	0.03	0.23	0.19	0.01
80th percentile income	−0.03	−0.03	−0.16	−0.35
20th percentile income	−0.04	−0.12	−0.15	−0.20
% Poverty	0.06	0.22	0.28	0.14

## Discussion

Most of the research investigating the course and impact of the COVID-19 pandemic in the United States has focused on the nation as a whole, or on individual states. Relatively less work has examined more localized effects of the pandemic. In addition, although prior research has established a clear link between poverty and the course of the disease (Adhikari, et al., 2020; Finch and Hernández Finch, 2020; Goldstein, 2020; Koma, et al., 2020), more nuanced investigations of this relationship have largely not been undertaken. Therefore, the goal of this study was to examine the course of the COVID-19 pandemic in the United States from winter 2020 through early summer 2021, and to ascertain whether there existed differential relationships between poverty level within individual counties and the number of deaths due to the virus over time. The results from the mixture multilevel model identified four distinct patterns based on the relationships of poverty and time with the number of COVID-19 deaths. In addition, the identified classes could also be distinguished based upon a number of demographic, income, and health care factors.

Given these findings that there was not a single COVID-19 pandemic within the United States, there are several implications for both policy makers, and researchers. First, it is clear that poverty is an important factor with respect to the number of COVID-19 deaths present in an American county, but that this relationship appears to be dependent to some extent on the level of urbanization and diversity present. In those counties which were largely rural and less diverse (latent classes 1 and 3), poverty was more weakly related to the number of COVID-19 deaths. In contrast, urban locations, particularly those in the latent Class 1 which were the most diverse, manifested stronger relationships between poverty and the COVID-19 death rate. More specifically, counties with higher proportions of residents who identified as white had lower death rates due to COVID-19 than those counties with a higher proportion of nonwhite individuals, and the relationship between poverty and the death rate was stronger in those counties with a greater share of nonwhite residents. This general divide between urban/more diverse and rural/less diverse counties was also clear in terms of the correlations between individual income and poverty variables with the number of COVID-19 cases present in the county. Thus, one challenge for policy makers going forward is to more fully investigate the public health response to COVID-19 and the availability of services for dealing with it within counties with higher proportions of nonwhite residents, and to take corrective measures to ensure that those regions have the resources necessary to deal with large scale health emergencies moving forward.

A second related implication of this study is that among urban locations, both the growth rate in the number of reported deaths, and the relationship between poverty and the number of deaths were largest in the most highly urbanized areas. Latent Classes 2 and 4 were comprised of the most urban counties in the United States, and also exhibited the fastest COVID-19 death growth rates and the strongest relationship between poverty and number of deaths. In addition, among the counties in those classes, those that were the most urbanized and densely populated (Class 4) had the fastest growth in the COVID-19 death rate and the strongest relationship between poverty and the death rate. Thus, policy makers and public health officials should not view all urban areas as the same when it comes to understanding and dealing with the spread of COVID-19. The largest and most densely populated urban centers in the United States had faster growth in the COVID-19 death rates and stronger relationships with poverty, than did the somewhat less populace urban counties, which comprised Class 2. And again, it should be noted that the counties in latent Class 1 were the most ethnically diverse, again highlighting the fact that even among urban counties, where the death rate was highest, those with greater proportions of nonwhite residents had even higher COVID-19 death rates. It is clear that race/ethnicity is an important factor in terms of the COVID-19 death rates across the United States

In contrast to the findings for urban areas, among the more rural counties (latent Classes 1 and 3) the relationship between poverty and the number of cases were quite different. In Class 1, which included the least diverse counties in the nation, the relationship between poverty and the rate of COVID-19 deaths was the weakest, particularly for the individual income and poverty variables. In contrast, the other more rural group (Class 3) exhibited somewhat a stronger relationship between the poverty index and the number of COVID-19 deaths, though still smaller than those exhibited by the two urbanized latent classes. These results once again point to the need for policy makers to consider the variegated nature of the pandemic across the country. Even among counties that shared similarities, such as being predominantly rural for example, there were different experiences of the pandemic both in terms of the raw death rates and its relationship with poverty. In addition, given that the counties in latent class 2 had larger proportions of nonwhite population than those in latent class 1, as well as higher death rates, policy makers must consider the role that ethnicity plays above and beyond poverty, when it comes to the impact of pandemics.

A fourth implication of these results is with respect to the pattern of death rates in the latent classes. These comparisons revealed that the two urbanized classes had the highest death rates per 1,000 residents, and the highest death rates per number of cases. Specifically, Class 4 had the fastest growth rate in the number of cases and the highest death rate per 1,000 residents, as well as the highest death rate per number of cases. The most rural and least diverse counties had the lowest death rates per 1,000 residents and per the number of COVID-19 cases. Of particular interest in this regard was the fact that the counties in Class 4 were collectively the wealthiest in the nation. And yet, they had the highest death rate and the strongest association between poverty and this rate. Given that these counties also had the highest proportion of nonwhite residents in the nation, there appear to be important policy implications regarding disparities in health care services for people of color. The question of the wealthiest counties in the nation, with the greatest access to high quality health care exhibited the highest death rates, and the greatest inequalities of that rate with respect to poverty must be addressed in future research.

A fifth implication of these differences in the course that the pandemic across the United States come in the form of challenges to policy makers and public health officials in terms of trying to craft a coherent and comprehensive policy that will work everywhere. As noted above, the pandemic in rural, predominantly white middle America was very different from the pandemic in highly diverse urban centers, which in turn experienced it differently than the somewhat smaller, poorer cities across the nation. And indeed, even among rural portions of America, the pandemic and associated death rates did not follow a uniform pattern, as seen in the different trajectories and relationships between poverty and COVID-19 deaths between the two primarily rural latent classes (latent Classes 1 and 3). In general, higher rates of poverty were associated with higher rates of death due to the disease, but this relationship was much more variegated than has been previously reported. Thus, moving forward, these results would suggest that the policies for dealing with the pandemic should also be more variegated and more directed than may have been the case heretofore.

### Limitations

As with any research, there are a number of limitations to the current study that need to be acknowledged, and which should offer opportunities for future work in this area. First, the model used in the study was fairly simple in nature. Only time and the poverty index were included as predictors. The reason for using this relatively limited model was to allow for a clear focus on the relationship between poverty and the rate of COVID-19 deaths, while accounting for changing course of the pandemic over time. However, there are certainly other variables, and other measures of poverty, which could be included in such an analysis, and which might yield different insights into the nature of the pandemic than those explored here. Thus, future work should expand upon this model by including other variables of interest in the mixture component of the analysis.

A second limitation of this research involves the variables used in the follow up analyses to the initial mixture model. The purpose of the follow-up was to more fully explore the nature of the latent classes as a way of gaining further insights into the interplay between poverty and the course of the pandemic. However, given the limitations of the manuscript length, as well as the desire to keep the results as clear and understandable as possible, some variables that would have been interesting to include were by necessity excluded. In particular, variables regarding health outcome rates for specific ethnic and income groups, as well as variables related to education and the trajectory of income and unemployment over the last 2 decades were not included in this study. It is believed that they might prove to be interesting in terms of further characterizing the latent classes, but given the aforementioned size limitations for the study, the decision was made to exclude them. Future research could pay special attention to these, and other variables, in an attempt to more fully understand the latent classes included in this work.

A third limitation of this study was that the data used in the analysis were at the county, rather than the individual level. Clearly, having person level data would be extremely informative in terms of understanding the nature of the COVID-19 pandemic, and its impact on individuals. However, such data is generally not available, and would likely need to be collected using a dedicated study design and sampling plan. Thus, although limited to some extent by the aggregated nature of the county level data, the current study does allow for an investigation that is nationwide. Nonetheless, future work should focus on specific regions or areas of the country with data at the individual level. This is a particularly important issue for measures of air pollution, which can vary locally within counties. Therefore, using a county level measure of particulate matter in the air at the county level is not ideal. Future work should attempt to obtain more locally specific information about air pollution to be included in the model.

A fourth limitation of the current study is the use of an ecological approach to the data analysis, in which data at the population level (the U.S. counties in this case) serve as the unit of analysis. Although confounding variables have been included in this study (e.g., measures of poverty, health care access, and employment), nuanced relationships among variables that would be possible for individual level data are not available with this population level data. Therefore, results of the multivariate modeling techniques, while informative and providing useful information, must be interpreted with the knowledge that these nuanced relationships cannot be fully explored with the current data structure.

Finally, as with any research focusing on a rapidly changing pandemic, the situation changes quickly. It is simply a reality of such research that changes in the situation on the ground will be continually occurring in such a fluid environment. Nonetheless, we believe that the results presented here provide researchers and policy makers with both a detailed description of the pandemic’s course in its first 9 months, as well as with information that can be used to guide future work in this area. It is clear that the pandemic in the United States cannot be viewed as a single event, particularly in the context of poverty. Thus, despite changes in case rates subsequent to the end of this work, the overall message remains relevant, namely that there is no single pandemic in the country, but rather that it manifests itself differently in different locales.

## Data Availability

Publicly available datasets were analyzed in this study. This data can be found here: https://www.nytimes.com/interactive/2020/us/coronavirus-us-cases.html.
