# Supplementary material for: Not One Pandemic: A Multilevel Mixture Model Investigation of the Relationship Between Poverty and the Course of the COVID-19 Pandemic Death Rate in the United States
Source: Front Sociol. 2021 Oct 22;6:629042. doi: 10.3389/fsoc.2021.629042 (PMC8570187; doi:10.3389/fsoc.2021.629042)
Supplement: Supplementary file 1 [file DataSheet1.docx]

Table S1: Model fit statistics by latent class

| Classes | AIC | BIC | aBIC | Entropy |
| --- | --- | --- | --- | --- |
| 2 | 1055990.02 | 1055733.17 | 1055690.92 | 0.881 |
| 3 | 1053362.76 | 1054107.24 | 1054098.00 | 0.884 |
| 4 | 1024955.78 | 1025197.51 | 1025114.89 | 0.902 |
| 5 | 1030422.65 | 1030962.23 | 1030871.44 | 0.893 |

Table S2: Average probabilities of most likely latent class

|  | 1 | 2 | 3 | 4 |
| --- | --- | --- | --- | --- |
| 1 | 0.998 | 0.000 | 0.002 | 0.044 |
| 2 | 0.000 | 0.957 | 0.030 | 0.013 |
| 3 | 0.002 | 0.083 | 0.915 | 0.000 |
| 4 | 0.000 | 0.035 | 0.000 | 0.964 |

Table S3: Mixed effects parameter estimates (standard error), proportion of counties in each latent class, mean poverty index, and ICC estimate for the four latent class solution

| Variable | Class 1 | Class 2 | Class 3 | Class 4 |
| --- | --- | --- | --- | --- |
| Proportion | 0.33 | 0.25 | 0.39 | 0.03 |
| Week coefficient | 0.05* (0.01) | 3.91* (0.14) | 0.84* (0.04) | 53.09* (1.72) |
| Poverty index coefficient | 0.03* (0.01) | 10.51* (0.62) | 0.40* (0.16) | 46.21* (10.04) |
| Poverty mean | 0.75 | -0.19 | -0.47 | 0.29 |
| ICC | 0.17 | 0.11 | 0.16 | 0.15 |

**p*<0.05

Table S4: Summary of ancillary variables by latent classes

|  | Class 1 |
| --- | --- |
| Model parameters | Slowest Covid-19 death growth rate; Weakest relationship between Covid-19 death rate and poverty. |
| Death rate | Had the lowest death rate per 1000 residents, and the lowest death rate per cases. |
| Demographics | Oldest; Least diverse |
| Health | Least access to exercise, lowest average daily particulate matter |
| Income | Lowest income |
| Housing/Food | Lowest housing cost burden and severe housing problems; highest homeownership; Next to lowest food insecurity |
| Urban/Rural | Most rural |
| Poverty | Little to no relationship between income or poverty rates with cases per 100,000 |
| Mobility |  |
|  | Class 2 |
| Model parameters | Second fastest Covid-19 death growth rate; Second strongest relationship between poverty index and Covid-19 rate. |
| Death rate | Second highest death rate per 1000 residents, and second highest death rate per cases. |
| Demographics | Second youngest; Second most diverse |
| Health | Second highest access to exercise; second lowest rates of smoking, age-adjusted deaths, lowest physical inactivity, lowest obesity, and diabetes |
| Income | Second highest income |
| Housing/Food | Second greatest housing burden, and second lowest homeownership rate; Second highest food insecurity. |
| Urban/Rural | Second most urban |
| Poverty | Strongest relationship between unemployment and death rates; Small, but not negligible relationships between % children enrolled in free lunch programs and % in Poverty with death rates. |
| Mobility | Second least change in mobility rates for transit stations and retail/recreation. Most change in mobility for parks, and second most for workplaces, residential, and grocery/pharmacy |
|  | Class 3 |
| Model parameters | Second slowest growth in Covid-19 death rate; Second weakest relationship between poverty and Covid-19 death rate. |
| Death rate | Had the second lowest death rate per 1000, and second lowest death rate per number of cases. |
| Demographics | Second oldest; second least diverse. |
| Health | Second lowest access to exercise rate; highest age-adjusted death rate and years of potential life lost. |
| Income | Lowest income similar to Class 1 |
| Housing/Food | Second lowest housing burden, and second highest homeownership; Most food insecure. |
| Urban/Rural | Second most rural |
| Poverty | Strongest relationships between death rates with % enrolled in free lunch and % in poverty. Second strongest relationships between death rates with income. |
| Mobility | Least change in mobility rates for transit stations, retail/recreation, workplaces, and grocery/pharmacy |
|  | Class 4 |
| Model parameters | Fastest Covid-19 death growth rate; Strongest relationship between poverty index and Covid-19 rate. |
| Death rate | Had the highest death rate per 1000 residents, and highest death rate per cases. |
| Demographics | Youngest; Most diverse |
| Health | Most access to exercise; lowest smoking rate; lowest age-adjusted death rate; lowest physical inactivity rate; lowest obesity and diabetes rates; highest daily particulate value |
| Income | Highest income |
| Housing/Food | Greatest housing burden, and lowest homeownership rate; Lowest food insecurity, and highest food environment index. |
| Urban/Rural | Most urban |
| Poverty | Strongest relationship between unemployment and case rates; Small, but not negligible relationships between income and case rates. |
| Mobility | Greatest change in mobility for transit stations, retail/recreation, workplaces, residential, and grocery/pharmacy. |

Figure 1: Map of Latent Class membership by county
